# Common strength and localization of spontaneous and evoked synaptic vesicle release sites

**DOI:** 10.1186/1756-6606-7-23

**Published:** 2014-04-02

**Authors:** Kristina Loy, Oliver Welzel, Johannes Kornhuber, Teja W Groemer

**Affiliations:** 1 Department of Psychiatry and Psychotherapy, Friedrich-Alexander-University of Erlangen-Nuremberg, Schwabachanlage 6, Erlangen 91054, Germany; 2 Present address: Institute of Clinical Neuroimmunology, Ludwig-Maximilians University Munich, Munich, Germany

## Abstract

**Background:**

Different pools and functions have recently been attributed to spontaneous and evoked vesicle release. Despite the well-established function of evoked release, the neuronal information transmission, the origin as well as the function of spontaneously fusing synaptic vesicles have remained elusive. Recently spontaneous release was found to e.g. regulate postsynaptic protein synthesis or has been linked to depressive disorder. Nevertheless the strength and cellular localization of this release form was neglected so far, which are both essential parameters in neuronal information processing.

**Findings:**

Here we show that the complete recycling pool can be turned over by spontaneous trafficking and that spontaneous fusion rates critically depend on the neuronal localization of the releasing synapse. Thereby, the distribution equals that of evoked release so that both findings demonstrate a uniform regulation of these fusion modes.

**Conclusions:**

In contrast to recent works, our results strengthen the assumption that identical vesicles are used for evoked and spontaneous release and extended the knowledge about spontaneous fusion with respect to its amount and cellular localization. Therefore our data supported the hypothesis of a regulatory role of spontaneous release in neuronal outgrowth and plasticity as neurites secrete neurotransmitters to initiate process outgrowth of a possible postsynaptic neuron to form a new synaptic connection.

## Findings

### Background

Central neurons display two different forms of vesicle release: stimulation dependent, i.e. evoked release and stimulation independent spontaneous release, which occurs at resting membrane potential. Spontaneous neurotransmitter release is thought to play a crucial role in synaptic plasticity, memory and learning [1] as well as in pathophysiology [2]. Furthermore spontaneous neurotransmitter release was found to regulate postsynaptic dendritic protein synthesis [3,4]. The origin of spontaneous release remains controversial, with some evidence arguing for the recycling pool [5,6] of vesicles and some for the reserve pool [7]. Here we seek to analyze the amount of spontaneous release regarding its turnover kinetics and cellular location. While a pool can be defined for each form of release, our results indicate a common regulation of both vesicle populations and thus a common origin of both pools from the recycling pool of vesicles.

## Results

**Figure 1:**
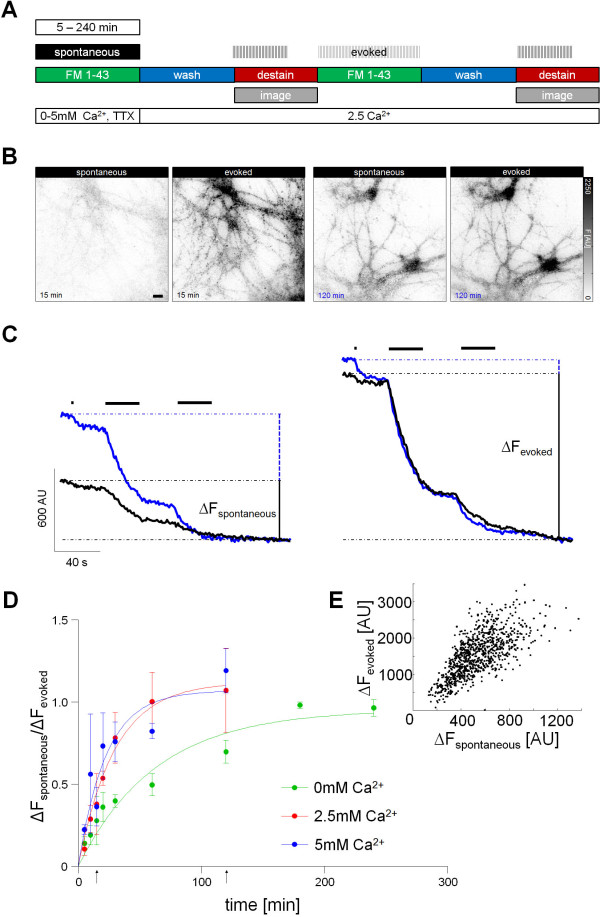
**The recycling pool of hippocampal synapses is turned over completely by spontaneous vesicle recycling. A** Scheme of experimental setup: Boutons were first labeled with FM1-43 by spontaneous uptake for different time periods. At the end of the first part of the experiment, boutons were completely destained using twice 900 pulses to determine the amount of spontaneous turnover. After a 10 minute recovery synaptic boutons were labeled a second time using 1200 electrically evoked action potentials and again completely destained to determine the recycling pool size. **B** Difference images for 15 and 120 minutes spontaneous FM1-43 uptake and evoked staining, respectively. Scale bar, 10 μm. **C** Corresponding mean fluorescence profiles to determine ∆F_spontaneous_ and ∆F_evoked_ for each time period. **D** Time course of ∆F_spontaneous_ to ∆F_evoked_ ratio depending on extracellular calcium concentration (*t*_*½*_ in minutes: 0 mM Ca^2+^ = 43.96, 2.5 mM Ca^2+^ = 20.58, 5 mM Ca^2+^ = 15.73). Arrows mark the exemplary time points. **E** Correlation of spontaneous and evoked release at individual synaptic boutons (time point at 60 min).

Using a live-cell imaging approach with the styryl dye FM1-43 we determined the amount of spontaneous and evoked release at individual hippocampal synapses. Therefore synapses underwent a spontaneous and an evoked round of staining, each followed by activity induced destaining (Figure 1A). For spontaneous staining incubation times of 5–240 minutes where used, depending on when saturation was reached. Calcium concentrations ranged from the total absence of calcium in the extracellular medium, physiological calcium concentrations (2.5 mM) to a high calcium concentration (5 mM). Using this paradigm we were able to quantify the recycling pool size as well as the spontaneously released vesicles after different loading times for spontaneously fusing vesicles. Additionally different calcium concentrations for spontaneous loading were tested. First we found that spontaneous release was able to turn over the complete recycling pool in a calcium dependent manner (Figure 1). Although the kinetics to reach saturation was slower at reduced calcium concentrations, all recycling vesicles were labeled at different concentrations of external calcium [8] (Figure 1D). Importantly [9], synapses that were capable of spontaneous vesicle release also underwent evoked vesicle recycling (97.87% ± 3.09% of boutons) and vice versa (98.61% ± 0.96% of boutons; Wilcoxon rank sum test: *p* = 0.83) and their spontaneous and evoked fluorescence changes were correlated (Figure 1E; Pearson’s *r* = 0.79 ± 0.06; 3 experiments, 921 boutons). To validate the turnover of the whole recycling pool by spontaneous release we used an alternative dual-color imaging approach with synapto-pHluorin (spH) transfected neurons and an αGFP-CypHer5E™ antibody to label the spontaneously fused vesicles. As both fluorescence markers were pH-sensitive with an inverse characteristic [10,11], which made them excellent tools for the analysis of exo- and endocytosis due to the acidic pH inside synaptic vesicles. In contrast to the previous FM1-43 experiments evoked and spontaneous release was measured simultaneously by quantifying the fluorescence decay of the αGFP-CypHer5E™ fluorescence signal, resembling the spontaneous labeled vesicles, and on the other hand the spH fluorescence increase, corresponding to activity dependent recycling pool of vesicles. In accordance to the FM1-43 experiments we demonstrated that the whole recycling pool, which corresponds to 37.10 ± 11.10% of the total pool, could be labeled (Additional file 1: Figure S1). In a next step we wanted to determine the fusion rate of a single spontaneously released vesicle to estimate the time to turn over the whole recycling pool of vesicles and thus to proof the kinetics described above. Therefore synapses were stained spontaneously with FM1-43 for only 15 minutes at zero calcium to label only a few vesicles and subsequently destained to monitor the resulting fluorescence decay (Additional file 1: Figure S2A). Analysis of these fluorescence changes using histograms exhibited a clear quantization that corresponds to intensity of a single vesicle, which enabled us to calculate the number of spontaneously fused vesicles during 15 minutes and thus the turnover rate of a single spontaneously fusing vesicle (Additional file 1: Figure S2B). Using this single vesicle quantification, we determined the spontaneous turnover rate to be one vesicle each 80.42 ± 10.77 seconds, which is in accordance with literature [5]. Assuming a recycling pool size of about 130 vesicles [11-13] and neglecting the effect of reuse of vesicles and the faster recycling kinetics of the RRP vesicles [14], this fusion rate resulted in a rough estimate of about 174 minutes to turn over the whole recycling pool by spontaneous fusion, which agrees with the time course described above. Furthermore similar to evoked release [14] spontaneous vesicles were instantly re-releasable and thus immediately repopulate the readily releasable pool (Additional file 1: Figure S2).

**Figure 2:**
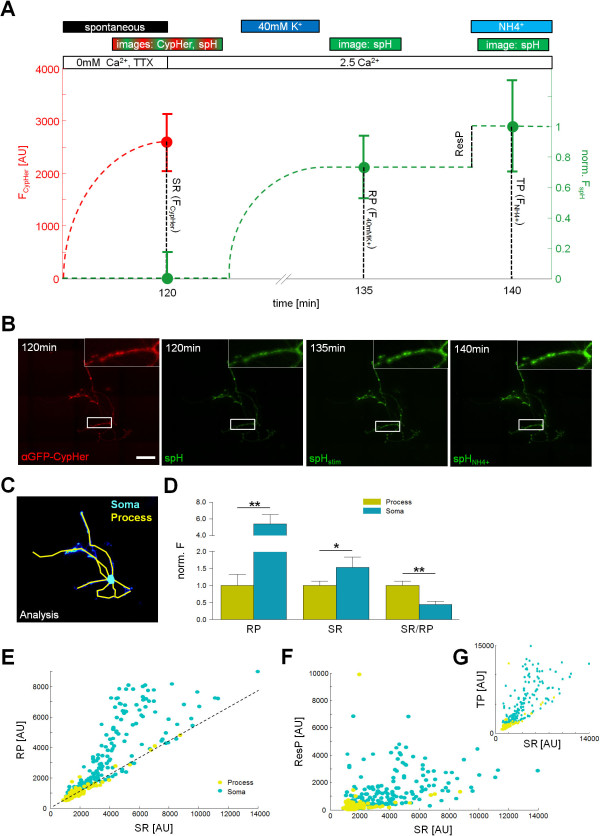
**At soma-near synapses the number of spontaneously fused vesicles as well the recycling pool vesicles is higher than in distal cellular compartments. A** Analysis of spontaneous and evoked release differentiated between process and soma using dual color experiments of spH transfected hippocampal neurons spontaneously labeled with αGFP-CypHer5E™. The spontaneous release (SR), recycling pool (RP), reserve pool (resP) and the total pool (TP) were determined. Scheme of experimental setup added with the mean values of spH and CypHer5E™ fluorescence used for further analysis. Boutons of spH transfected hippocampal neurons were first labeled with αGFP-CypHer5E™ by spontaneous uptake during a 120 minute period. Then a stitched image was captured to encompass the whole neuron with its processes at high resolution in both fluorescence channels (120 min). After stimulation with 40 mM K^+^ solution for 2 minutes in the presence of Bafilomycin an image of the same region was recorded (135 min), followed by an image after NH_4_^+^ application (140 min). **B** Images depicting whole neuron. Insets: Synaptic boutons at higher magnification. Scale bar, 50 μm. **C** Scheme of image analysis used for distinction between process and soma. **D** Quantification of pool sizes: RP (two-sample *t*-test: *p* = 0.001), SR (two-sample *t*-test: *p* = 0.042) and SR to RP ratio (SR/RP; two-sample *t*-test: *p* = 0.005; 11 experiments). Correlations of distinct vesicle pools differentiated for synapses located at the process or the soma: SR and RP (**E**; Pearson’s *r*_*process*_ = 0.96 ± 0.01, *r*_*soma*_ = 0.84 ± 0.04), SR and ResP (**F**; Pearson’s *r*_*process*_ = 0.19 ± 0.14, *r*_*soma*_ = 0.51 ± 0.11) and SR and TP (**G**; Pearson’s *r*_*process*_ = 0.64 ± 0.09, *r*_*soma*_ = 0.78 ± 0.05). Dashed line in **E** represents the linear fit of a subset of data putative resembling the processes running above or beneath the soma (slope_process_ = 0.55 ± 0.02, fit: *y = mx + t*).

In order to determine the strength of spontaneous release at the soma and the neuronal processes, we adopted a dual-color imaging approach with spH transfected neurons and an αGFP-CypHer5E™ antibody to label spontaneously fused synaptic vesicles (Figure 2A, B). Additionally we made use of a low transfection rate and chose neurons that fluoresced isolated among untransfected neighboring neurons for the analysis. The soma and the neurites were set manually (Figure 2C). Sholl analysis revealed a normal branching pattern (Σ_spH_ = 1.88 ± 0.34; Σ_mCherry_ = 1.26 ± 0.33) [15]. We found that the size of the recycling pool as well as the number of spontaneously fused vesicles is higher at the soma than in distal cellular compartments indicating a higher absolute release. However, the relative amount of spontaneous turnover, normalized on the recycling pool size, was significantly higher in the periphery (Figure 2D). Therefore the smaller synapses in the periphery have a lower absolute spontaneous turnover, but release relative to their recycling pool more vesicles spontaneously. Distinction of the axon, the dendrites and the soma via MAP2 immunostaining also showed that in absolute values synapses near the soma released the highest number of spontaneous vesicles during a 120 minute period (Additional file 1: Figure S3). The fact that synapses located at the dendrite released more spontaneous vesicles compared to the axon can be explained by the shorter length of the dendrite and thus their closer average proximity to the soma. These results fit to the notion that vesicle recycling is more effective at smaller synapses [11] arguing for neuronal outgrowth in distal segments. Spontaneous release showed a strong correlation with the size of recycling and total pool at neurites and soma, respectively (Figure 2E, G). On the other hand, no clear relationship between spontaneous release and the reserve pool was observed (Figure 2F). The fact that spontaneous release correlates with the size of recycling and total pool, but not with the size of the reserve pool seems controversial. This can be due to the high variability of the pool sizes [16] especially the reserve pool [17] and heterogeneous release probabilities among synapses [18]. Similar to evoked release, the frequency distribution of spontaneous release is more right-skewed at the soma when compared to the process (Additional file 1: Figure S4A-C), which is consistent with more recycling vesicles at the soma-near synapses (Figure 2D). No differences between synapses at the dendrite and the axon could be observed (Additional file 1: Figure S4D). Synapses were then pooled and sorted into the 20% largest, smallest and the 20% around the medium size. Regarding these different synapse sizes and locations of individual boutons, these diverse distributions caused distinct fractions of the recycling and reserve pool size with respect to the total pool size (Additional file 1: Figure S4E). In absolute numbers spontaneous release declined with distance to the soma (Additional file 1: Figure S5A), but remained largely constant if spontaneous turn-over was normalized on synapse size (Additional file 1: Figure S5B).

## Conclusion and discussion

We provide new evidence that spontaneous vesicle turnover can reach the level of the recycling pool of vesicles in a time and calcium dependent manner (Figure 1D, Additional file 1: Figure S1C). Considering these results together with the fact that the amount of recycling pool vesicles correlates robustly with the spontaneous release at each bouton (Figure 1E), suggest that spontaneous vesicles originate from the recycling pool of vesicles rather than from the reserve pool. This underlines previous findings [5,6,19], but is contrary to studies that pointed at the reserve pool as origin of spontaneous vesicles [7]. These previous studies used a sequential labeling paradigm instead of a simultaneous labeling that we used in our study. We cannot exclude with our experimental approach that, after total recycling pool turnover, additional vesicles recycle spontaneously from within the reserve pool of vesicles, due to a lack of discrimination without sequential labeling after saturation. However such vesicles would account for a minority of spontaneously fusing vesicles due to the lack of correlation with the reserve pool (Figure 2F). Besides we found that the soma has absolutely the larger synapses with the larger recycling pool and the higher spontaneous release, but if spontaneous and evoked turnover is normalized on the size of the synapse, the relative release is higher at the processes. Recent publications found a distance from soma dependency of synapse size and evoked release at the processes [20,21]. In accordance with evoked release [21], spontaneous release declined along the processes with increasing distance to the soma (Additional file 1: Figure S5A), but remained constant, if spontaneous release was normalized on synapse size (Additional file 1: Figure S5B). Our functional measurements indicated, that both forms of release exhibit the same relationship regarding distance from the soma with smaller, but more effective synapses at the process [11]. These results therefore point to a common developmental origin of these release modes with the vesicle populations stemming both from the recycling pool of vesicles. We also found differences between soma and processes regarding synapse size, relative and absolute release and confirmed, that smaller synapses release more efficiently. In conclusion we found a multitude of commonalities of spontaneous release and evoked release, e.g. correlation and identical size with recycling pool, vesicles were immediately re-releasable, same cellular localization with respect to release characteristics which together suggests the recycling pool as the common source of spontaneous and evoked released vesicles. Nevertheless a definition of a spontaneous vesicle pool is valid as vesicles differ with respect to their neuronal function and more future research is needed to substantiate the origin of spontaneous vesicles and to further address the function of these vesicles.

## Competing interests

The authors declare no competing financial interests.

## Authors’ contributions

KL, OW designed and performed experiments. KL, OW and TWG evaluated and wrote the manuscript. JK provided intellectual input. All authors read and approved the final manuscript.

## Supplementary Material

Additional file 1: Figure S1 Validation of FM1-43 experiments using spH transfected neurons and an αGFP-CypHer5E™ antibody. **Figure S2.** Single vesicle release after spontaneous FM1-43 staining. **Figure S3.** Analysis of spontaneous release at the soma, the axon and the dendrite. **Figure S4.** Pool size distribution at the soma and the processes. **Figure S5.** Relationship between the distance from the soma and spontaneous release. Click here for file

## References

[B1] Emptage NJ Reid CA Fine A Calcium stores in hippocampal synaptic boutons mediate short-term plasticity, store-operated Ca2+ entry, and spontaneous transmitter release Neuron 2001 29 197 208 10.1016/S0896-6273(01)00190-8 11182091

[B2] Autry AE Adachi M Nosyreva E Na ES Los MF Cheng PF Kavalali ET Monteggia LM NMDA receptor blockade at rest triggers rapid behavioural antidepressant responses Nature 2011 475 91 95 10.1038/nature10130 21677641 PMC3172695

[B3] Sutton MA Taylor AM Ito HT Pham A Schuman EM Postsynaptic decoding of neural activity: eEF2 as a biochemical sensor coupling miniature synaptic transmission to local protein synthesis Neuron 2007 55 648 661 10.1016/j.neuron.2007.07.030 17698016

[B4] Sutton MA Wall NR Aakalu GN Schuman EM Regulation of dendritic protein synthesis by miniature synaptic events Science 2004 304 1979 1983 10.1126/science.1096202 15218151

[B5] Hua Y Sinha R Martineau M Kahms M Klingauf J A common origin of synaptic vesicles undergoing evoked and spontaneous fusion Nat Neurosci 2010 13 1451 1453 10.1038/nn.2695 21102448

[B6] Groemer TW Klingauf J Synaptic vesicles recycling spontaneously and during activity belong to the same vesicle pool Nat Neurosci 2007 10 145 147 10.1038/nn1831 17220885

[B7] Fredj NB Burrone J A resting pool of vesicles is responsible for spontaneous vesicle fusion at the synapse Nat Neurosci 2009 12 751 758 10.1038/nn.2317 19430474 PMC2738656

[B8] Marra V Burden JJ Thorpe JR Smith IT Smith SL Hausser M Branco T Staras K A preferentially segregated recycling vesicle pool of limited size supports neurotransmission in native central synapses Neuron 2012 76 579 589 10.1016/j.neuron.2012.08.042 23141069 PMC3526798

[B9] Kavalali ET Chung C Khvotchev M Leitz J Nosyreva E Raingo J Ramirez DM Spontaneous neurotransmission: an independent pathway for neuronal signaling? Physiology (Bethesda) 2011 26 45 53 10.1152/physiol.00040.2010 21357902

[B10] Miesenbock G De Angelis DA Rothman JE Visualizing secretion and synaptic transmission with pH-sensitive green fluorescent proteins Nature 1998 394 192 195 10.1038/28190 9671304

[B11] Welzel O Henkel AW Stroebel AM Jung J Tischbirek CH Ebert K Kornhuber J Rizzoli SO Groemer TW Systematic heterogeneity of fractional vesicle pool sizes and release rates of hippocampal synapses Biophys J 2011 100 593 601 10.1016/j.bpj.2010.12.3706 21281573 PMC3030169

[B12] Branco T Staras K Darcy KJ Goda Y Local dendritic activity sets release probability at hippocampal synapses Neuron 2008 59 475 485 10.1016/j.neuron.2008.07.006 18701072 PMC6390949

[B13] Ryan TA Reuter H Smith SJ Optical detection of a quantal presynaptic membrane turnover Nature 1997 388 478 482 10.1038/41335 9242407

[B14] Pyle JL Kavalali ET Piedras-Renteria ES Tsien RW Rapid reuse of readily releasable pool vesicles at hippocampal synapses Neuron 2000 28 221 231 10.1016/S0896-6273(00)00098-2 11086996

[B15] Sholl DA Dendritic organization in the neurons of the visual and motor cortices of the cat J Anat 1953 87 387 406 13117757 PMC1244622

[B16] Moulder KL Jiang X Taylor AA Shin W Gillis KD Mennerick S Vesicle pool heterogeneity at hippocampal glutamate and GABA synapses J Neurosci 2007 27 9846 9854 10.1523/JNEUROSCI.2803-07.2007 17855599 PMC6672647

[B17] Branco T Marra V Staras K Examining size-strength relationships at hippocampal synapses using an ultrastructural measurement of synaptic release probability J Struct Biol 2010 172 203 210 10.1016/j.jsb.2009.10.014 19895891 PMC3084449

[B18] Murthy VN Sejnowski TJ Stevens CF Heterogeneous release properties of visualized individual hippocampal synapses Neuron 1997 18 599 612 10.1016/S0896-6273(00)80301-3 9136769

[B19] Wilhelm BG Groemer TW Rizzoli SO The same synaptic vesicles drive active and spontaneous release Nat Neurosci 2010 13 1454 1456 10.1038/nn.2690 21102450

[B20] Peng X Parsons TD Balice-Gordon RJ Determinants of synaptic strength vary across an axon arbor J Neurophysiol 2012 107 2430 2441 10.1152/jn.00615.2011 22279193 PMC3362249

[B21] de Jong AP Schmitz SK Toonen RF Verhage M Dendritic position is a major determinant of presynaptic strength J Cell Biol 2012 197 327 337 10.1083/jcb.201112135 22492722 PMC3328377

