# Supplementary material for: Common strength and localization of spontaneous and evoked synaptic vesicle release sites
Source: Mol Brain. 2014 Apr 2;7:23. doi: 10.1186/1756-6606-7-23 (PMC4022376; doi:10.1186/1756-6606-7-23)
Supplement: Additional file 1: Figure S1 — Validation of FM1-43 experiments using spH transfected neurons and an αGFP-CypHer5E™ antibody. Figure S2. Single vesicle release after spontaneous FM1-43 staining. Figure S3. Analysis of spontaneous release at the soma, the axon and the dendrite. Figure S4. Pool size distribution at the soma and the processes. Figure S5. Relationship between the distance from the soma and spontaneous release. [file 1756-6606-7-23-S1.docx]

**Additional file**

**Common strength and localization of spontaneous and evoked synaptic vesicle release sites**

Kristina Loy^*1,2^, Oliver Welzel^*1,^, Johannes Kornhuber^1^, and Teja W. Groemer^§1^

^1^Department of Psychiatry and Psychotherapy, Friedrich-Alexander-University of Erlangen-Nuremberg, Schwabachanlage 6, 91054 Erlangen, Germany

^2^present address: Institute of Clinical Neuroimmunology, Ludwig-Maximilians University Munich, Munich, Germany.

^§^ *Correspondence should be addressed to*:

Dr. Teja W. Groemer

Phone: +49 9131 85 44781

Fax: +49 9131 85 36381

E-mail: [teja.groemer@uk-erlangen.de](mailto:teja.groemer@uk-erlangen.de)

^*^ These authors contributed equally to this work

Supplementary methods: 1659 words

Supplementary figures: 619 words

**Supplementary Materials and methods**

***Cell culture and transfection***

Hippocampal neuronal cultures were prepared from 1- to 4-day-old Wistar rats. Briefly, newborn rats were sacrificed by decapitation in accordance with the guidelines of the State of Bavaria. The hippocampi were removed from each brain and were transferred into ice-cold Hank’s salt solution, and the dentate gyrus of each was cut away. After digestion with trypsin (5 mg ml^-1^), cells were triturated mechanically and plated in MEM, supplemented with 10% fetal calf serum and 2% B27 Supplement (all from Invitrogen, Taufkirchen, Germany). If required, neurons were transfected on DIV3 (days in vitro) with synapto-pHluorin [[1](#_ENREF_1)] (spH) or mCherry under the control of a synapsin promoter with a modified calcium phosphate method [[2](#_ENREF_2)]. Experiments were performed between DIV20 and DIV30.

***Imaging***

Experiments were conducted at room temperature on a Nikon TI-Eclipse inverted microscope equipped with a 60x, 1.2 NA water immersion objective and Perfect Focus System™. Fluorescent dyes were excited by a Nikon Intensilight C-HGFI through excitation filters at 472/30nm, 562/40nm and 628/40nm using dichroic longpass mirrors (cut-off wavelength 495nm, 593nm and 660nm). The emitted light passed emission band-pass filters at 520/35nm, 641/75nm and 692/40nm (Semrock, Rochester, USA) and was projected onto a cooled EM-CCD camera (iXon^EM^ DU-885 or DU-897, Andor, Belfast, Ireland).

Cover slips were placed into a perfusion chamber (volume = 500μl) filled with 2.5mM Ca^2+^ SBM. Synaptic boutons were stimulated by electric field stimulation (platinum electrodes, 10mm spacing, 1ms pulses of 50mA, 36V, and alternating polarity); 10µM 6-cyano-7-nitroquinoxaline-2,3-dione (CNQX, Tocris Bioscience, Bristol, UK) and 50µM D-amino-5-phosphonovaleric acid (D,L-AP5, Tocris Bioscience) were added to prevent recurrent activity.

***FM dye experiments***

Recycling synaptic vesicles were labeled spontaneously with 5 µM FM 1-43 (Invitrogen) at different calcium concentrations and in the presence of 1µM TTX (Sigma-Aldrich, Taufkirchen, Germany) for different time spans between 5 and 240 minutes. The used extracellular media were as following: 0mM Ca^2+^ SBM (in mM: 144 NaCl, 2.5 KCl, 5 MgCl_2_, 10 glucose, 10 Hepes, pH=7.4); 2.5mM Ca^2+^ SBM (in mM: 144 NaCl, 2.5 KCl, 2.5 CaCl_2_, 2.5 MgCl_2_, 10 Glucose, 10 Hepes, pH 7.4); 5mM Ca^2+^ SBM (in mM: 144 NaCl, 2.5 KCl, 5 CaCl2, 10 glucose, 10 Hepes, pH=7.4). After thorough washout of external dye cells were destained with 40 pulses at 20Hz to determine the size of the readily releasable pool, and twice with 900 pulses at 30Hz in 2.5mM Ca^2+^ SBM to destain all labeled vesicles. Then cells were stained a second time with FM1-43 using 1200 pulses at 30Hz to label the whole recycling pool. After extracellular dye wash out cells were destained and imaged as described before. The measured fluorescence change after spontaneous labeling was normalized on the fluorescence change after evoked loading, to obtain the relative fraction of spontaneous labeled vesicles of whole recycling pool.

For the detection of single vesicle exocytosis cells were stained with 10μM FM1-43 in 0mM Ca^2+^ SBM with 1μM TTX for 15 minutes in the incubator. After intensive dye washout cells were placed on the microscope and destained with 40 pulses at 20 Hz, followed by twice 900 pulses at 30 Hz in 2.5mM Ca^2+^ SBM. To determine the fluorescence of a single synaptic vesicle, we applied multi-Gaussian fits to the fluorescence intensity histogram of each experiment, which showed a clear quantal distribution (Fig.S2). The histograms were built from the fluorescence changes induced by 40 pulses at 20 Hz, which released vesicles from the readily releasable pool. Note that only a fraction of these vesicles are labeled that is to say those, which were stained during the 15 minutes period and repopulated the readily releasable pool. The spontaneous fusion rate was then calculated by division of the incubation time by the total number of released vesicles. The total number of stained vesicles during the 15 minutes period was obtained by summation of the fluorescence changes induced by all there stimulations and division by the intensity of a single vesicle.

***CypHer5E^TM^ experiments***

spH transfected cells were labeled for 30 minutes up to 240 minutes in the incubator in 0mM Ca^2+^ SBM with 1μM TTX with αGFP-CypHer5E^TM^. Cells were washed intensively in normal Ca^2+^ SBM with 80nM Concanamycin A (Sigma-Aldrich) to inhibit re-acidification of vesicles. Then cells were stimulated with 40 pulses at 20 Hz followed by 900 pulses at 30 Hz. This experimental paradigm provides values for the size of the readily releasable pool, the recycling pool and the spontaneous turnover which occurred during incubation (Fig.S1B). At the end of the protocol the SBM in the perfusion chamber was replaced for NH_4_^+^ SBM to determine the size of the total pool by unquenching of the maximal spH fluorescence. The reserve pool size was calculated by subtracting the size of the recycling pool from the total pool size. Individual CypHer5E^TM^ fluorescence profiles were normalized to their corresponding total pool fluorescence.

***Stitched images***

Stitched images were acquired with 5x5 fields in x and y direction and a 21 stage z-stack via the menu “Large Images” in NIS-Elements (Nikon Instruments Europe B.V., Badhoevedorp, The Netherlands). Stitching and a maximum intensity projection were automatically done by NIS-Elements. spH transfected cells were labeled spontaneously with an αGFP-CypHer5E^TM^ antibody for 120 minutes with 1µM TTX and 0mM Ca^2+^ SBM at 37°C. Using of a low transfection rate caused neurons that fluoresced isolated among untransfected neighboring neurons, which were chosen for the analysis and ensured that all spontaneous antibody labeling is related to one neuron. The SBM was then exchanged for high K^+^ SBM (in mM: 106.5 NaCl, 40 KCl, 10 glucose, 10 Hepes, 2.5 Mg^2+^, 2.5 Ca^2+^, pH=7.4) and 1μM Bafilomycin A1 (Merck KGaA, Darmstadt, Germany) and left for two minutes to turn over the whole recycling pool [[3](#_ENREF_3)]. Afterwards a large image of spH fluorescence was taken of the same region. To obtain a measure of the entire vesicle pool by direct alkalization the SBM was next changed for NH_4_^+^ SBM (in mM: 94 NaCl, 2.5 KCl, 10 glucose, 10 Hepes, 2.5 Ca^2+^, 2.5 Mg^2+^, 50 NH4Cl, pH=7.4).

***Immunofluorescence***

Hippocampal neurons were transfected with mCherry under the synapsin promotor (psyn-mCherry) and labeled spontaneously with αSynaptotagmin-Alexa488 (αSYT-Alexa488; SYSY, Göttingen, Germany; 1:100) for 120 minutes at 37°C in 0mM Ca^2+^ SBM with 1µM TTX. For MAP2 (Microtubule-associated protein 2) immunostaining, psyn-mCherry transfected cells were fixed in 4% Roti-Histofix (Carl Roth, Karlsruhe, Germany), permeabilized in 0.1% Triton in Roti-Immunoblock (Carl Roth) and blocked for 1 h in Roti-Immunoblock. Afterwards cells were first exposed to a MAP2 antibody (Merck KGaA; 1:1000) and subsequently to a goat-α-rabbit-Cy5 antibody (Invitrogen; dilution 1:500). Coverslips were mounted on slides with cytomounting medium (Dako, Hamburg, Germany). The different labelings served different purposes. The mCherry transfection was used to identify solely lying neurons in a network of untransfected neurons. Discrimination of axons and dendrites was possible with the MAP2 immunolabeling, selectively staining only dendrites. The spontaneous turnover was measured by the Alexa488 fluorescence as only vesicles were labeled that recycled spontaneously during 120 minutes.

***Data analysis***

All image and data analysis was performed using custom-written routines in MATLAB (The MathWorks, Inc., Natick, USA). Image stacks were used to automatically define regions of interest of synaptic bouton size [[4](#_ENREF_4)], where stimulation-evoked fluorescence increases or decreases occurred. The absolute fluorescence change of each bouton was calculated as the difference of the mean of at least three values before the onset of the respective stimulation and after the end of the stimulus. For the evaluation of synapse sizes and respective pool distributions all previously detected synapses of the respective group were pooled and then separated into the 20% smallest, largest and the 20% around the median, according to their total pool size [[5](#_ENREF_5)].

The half-life *t_½_* in minutes of a single exponential function was estimated using a least squared error fit to data points shown in Fig.1 and Fig.S1 using GraphPad® Prism 5.04 (GraphPad Software, Inc., La Jolla, USA). Linear fit (Fig.2) was conducted using MATLAB. In the analysis of the relationship between the distance from the soma and the strength of spontaneous release (Fig.S5) synapses were grouped in equally sized bins and averaged. Distance measurement was also done by a custom-written MATLAB routine and the obtained results were independent of the number of bins.

For the analysis of synapse and pool size distribution at the soma and the processes the fluorescence intensities were normalized using the equation:

$f_{n}=(f-\mu)/\sigma$,

whereas $\mu=\frac{1}{n}\sum_{i=1}^{n} f_{i}$ and $\sigma=\sqrt{\sum_{i=1}^{n} {(f_{i}-\mu)}^{2}}$. This normalization was indispensable for statistical comparison of the distributions. Resulting values $f_{n}$ were used to generate histograms.

For the correlation analysis, the Pearson’s correlation coefficient *r* or Spearman’s rank correlation coefficient *ρ* was calculated, respectively.

Dependent on the sample size and on the respective distribution, statistical comparisons were made with two-sample *t*-tests, one-way ANOVAs, Wilcoxon rank sum test or a Kruskal-Wallis test, respectively. If necessary Tukey’s post hoc test was applied. Distributions were compared using a two-sample Kolmogorov-Smirnov test. Error bars and specified values indicate standard error of the mean (SEM) if not stated otherwise. Levels of significance *p* (*p*-values) are indicated as follows: *ns*=not significant, * *p*<0.05, ** *p*<0.01 and *** *p*<0.001.

For the distinction between the soma and the processes, namely the axon and the dendrite, of a single neuron a semi-automatic analysis were conducted. Therefore the processes and the soma of a solitary neuron, which was transfected with either spH or mCherry, were traced or rather marked manually. If required the axon was selected on the basis of the MAP2 immunostaining. In a next step the synaptic boutons were detected automatically as described above and were assigned to the corresponding morphological structure. Furthermore the distance from the soma was determined automatically for each bouton located at a process.

Sholl analysis was performed using an ImageJ plugin (Fiji; Sholl Analysis Plugin (v1.0); http://labs.biology.ucsd.edu/ghosh/software/) which was downloaded from the website of the Gosh Lab. Large Images from the spH transfected neurons and the mCherry transfected neurons were analyzed and step radius was defined as 10μm. Σ represents the number of branches per 10µm.

Graphs were generated using MATLAB or GraphPad Prism® 5.04.

**References**

1. Sankaranarayanan S, De Angelis D, Rothman JE, Ryan TA: **The use of pHluorins for optical measurements of presynaptic activity.** *Biophys J* 2000, **79:**2199-2208.

2. Threadgill R, Bobb K, Ghosh A: **Regulation of dendritic growth and remodeling by Rho, Rac, and Cdc42.** *Neuron* 1997, **19:**625-634.

3. Ryan TA, Reuter H, Wendland B, Schweizer FE, Tsien RW, Smith SJ: **The kinetics of synaptic vesicle recycling measured at single presynaptic boutons.** *Neuron* 1993, **11:**713-724.

4. Sbalzarini IF, Koumoutsakos P: **Feature point tracking and trajectory analysis for video imaging in cell biology.** *J Struct Biol* 2005, **151:**182-195.

5. Welzel O, Tischbirek CH, Jung J, Kohler EM, Svetlitchny A, Henkel AW, Kornhuber J, Groemer TW: **Synapse clusters are preferentially formed by synapses with large recycling pool sizes.** *PLoS One* 2010, **5:**e13514.

**Supplementary Figures**


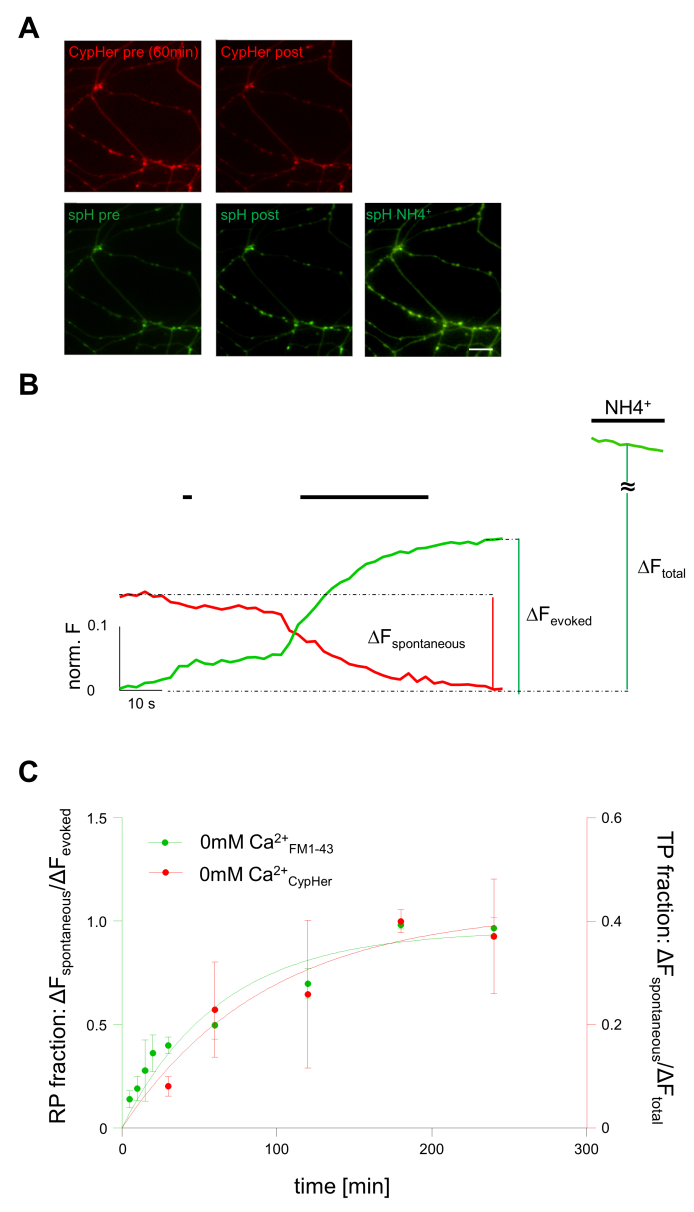


Fig.S1: Validation of FM1-43 experiments using spH transfected neurons and an αGFP-CypHer5E^TM^ antibody. **A** Exemplary images of dual color experiments. Boutons of spH transfected neurons were labeled with αGFP-CypHer5E^TM^ by spontaneous uptake during a 30 to 240 minutes period (pre). Then neurons were first stimulated with 40 pulses at 20Hz followed by a stimulus of 900 pulses at 30Hz as a measure for whole recycling pool in the presence of Concanamycin A1 to inhibit re-acidification of vesicles (post). At the end NH_4_^+^ was added to obtain a measurement for the total pool (NH_4_^+^). Thus the CypHer5E^TM^ signal is a measure for the spontaneous uptake, whereas the spH signal is a measure for the recycling and the total pool, respectively. Scale bar, 10µm. **B** Mean fluorescence profiles to **A** for the determination of ∆F_spontaneous_, ∆F_evoked_ and ∆F_total_ respectively. **C** Time course of ∆F_spontaneous_ to ∆F_total_ ratio at 0mM Ca^2+^ and corresponding FM1-43 time course as in Fig.1D for comparison (*t_½_* in minutes: 0mM Ca^2+^_FM1-43_=43.96, 0mM Ca^2+^_antibody_=64.50).


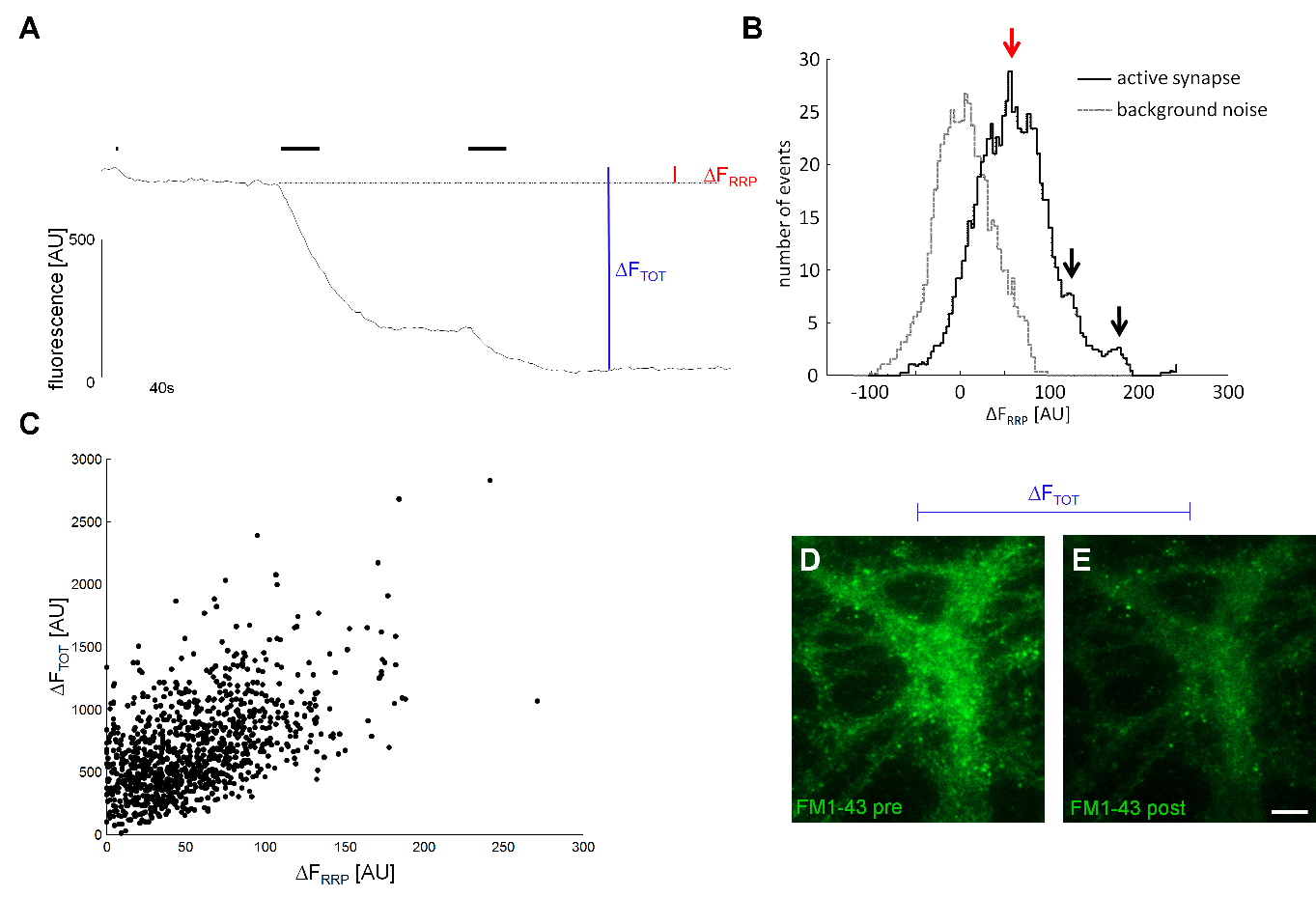


Fig.S2: Single vesicle release after spontaneous FM1-43 staining. **A**  Mean fluorescence intensity profile of spontaneously labeled vesicles. The first stimulation represents a train of 40 pulses at 20Hz, the second and the third represent 900 pulses at 30Hz. ∆F_RRP_ represents fluorescence loss by exocytosis induced by the RRP stimulus of 40 pulses at 20Hz and ∆F_TOT_ represents fluorescence destaining of all formerly spontaneously labeled vesicles. **B** Histogram of ∆F_RRP_ intensity distribution. The maximum at 67.38 AU marks the intensity of a single vesicle (red arrow); multiple vesicles released are indicated by the black arrows. Distribution of background fluorescence is marked in grey. **C** Correlation between total fluorescence change ∆F_TOT_ and RRP related fluorescence change ∆F_RRP_ (Pearson’s *r*=0.51752, *p*<0.001; 3 experiments). **D** and **E** Representative FM1-43 images before and after stimulation. Scale bar, 10µm.


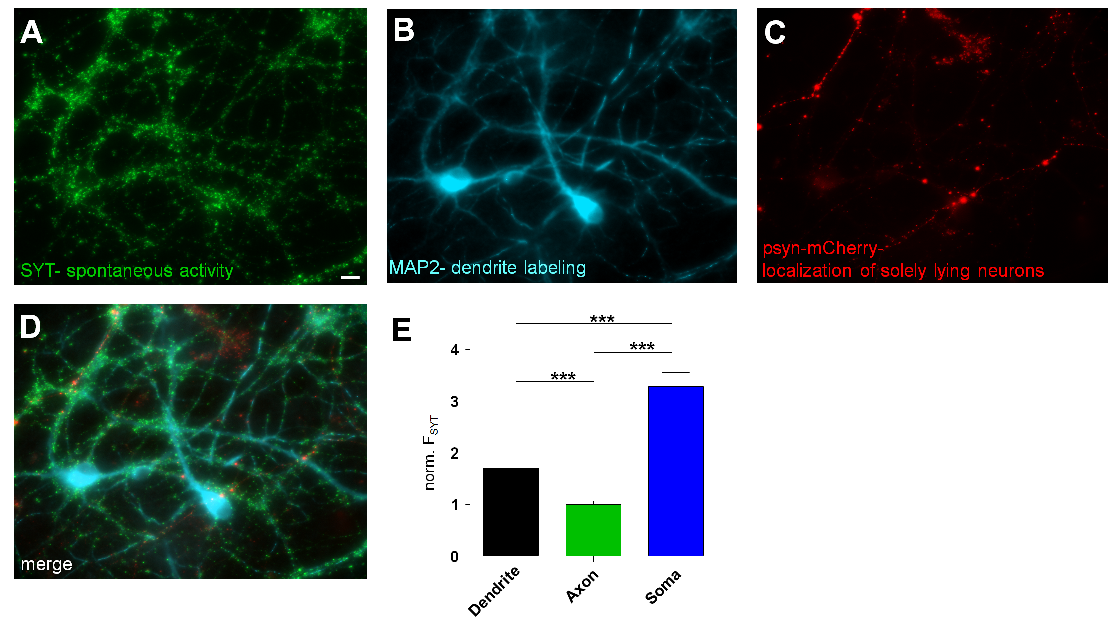


Fig.S3: Analysis of spontaneous release at the soma, the axon and the dendrite. **A-E** Illustrative images of stained hippocampal neurons. The intention of the different labeling is indicated in the pictures. Scale bar, 10µm. **A** Spontaneous labeling with an αSynaptotagmin (SYT)-Alexa488 antibody. **B** Determination of dendritic processes with an αMAP2-Cy5 (MAP2) immunofluorescence. **C** Neurons were transfected with mCherry under the synapsin promoter (psyn-mCherry) to visualize processes of solely lying neurons. **D** Overlay of all channels. **E** Mean fluorescence intensities of the spontaneously labeled release analyzed as the absolute Alexa488 fluorescence and normalized on the mean value of the axon (one-way ANOVA: *F*_2,2021_ = 512.2; *p*<0.0001; Tukey’s post hoc test: *p*<0.001; 14 experiments).

**
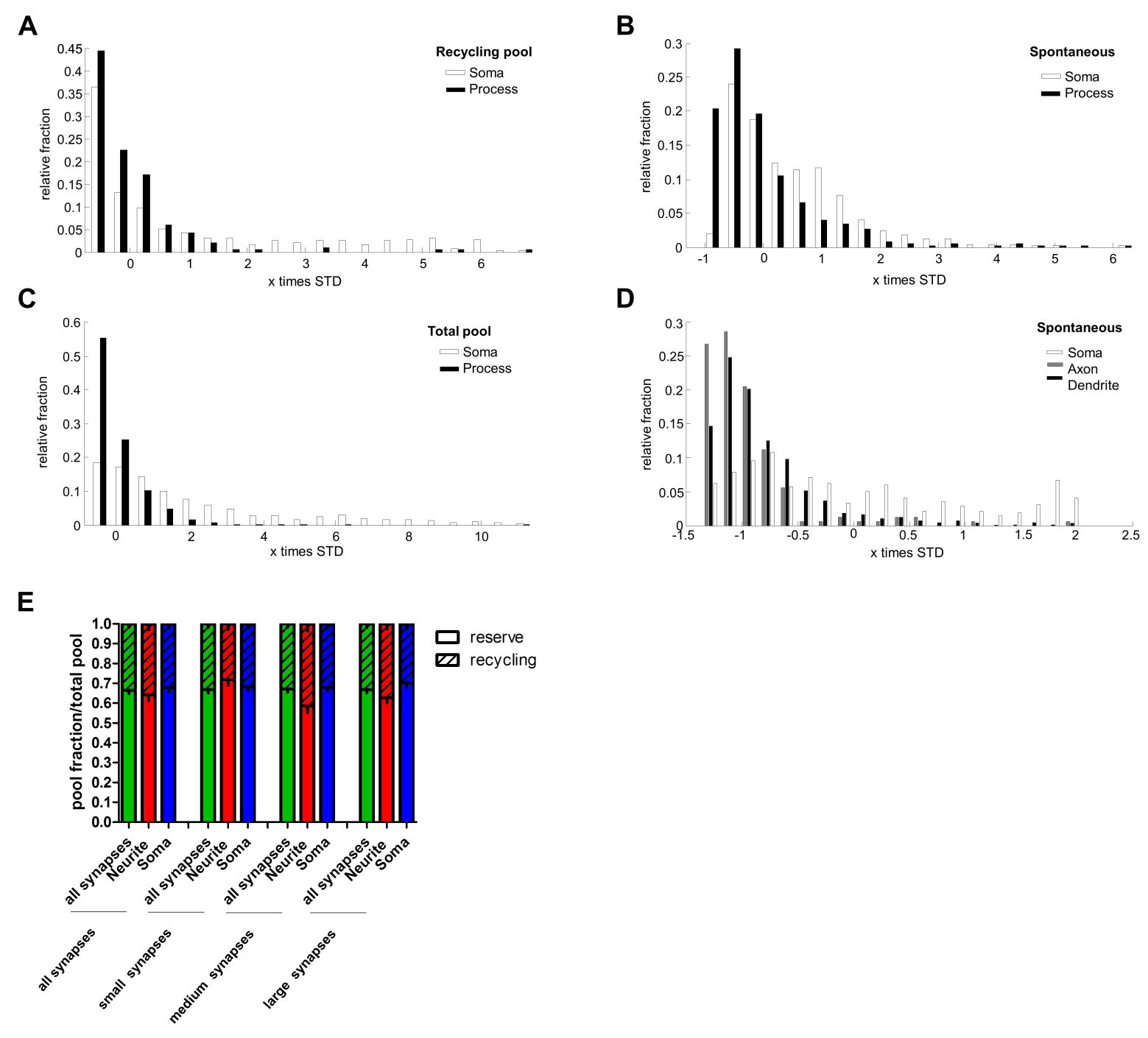
**

Fig.S4: Pool size distribution at the soma and the processes. Distributions of recycling pool sizes (**A**) or spontaneous release (**B**) at the process and the soma (for both experiments: two-sample Kolmogorov-Smirnov test: *p*<0.001). **C** Histogram of total pool size distribution (two-sample Kolmogorov-Smirnov test: *p*<0.001). **D** Histogram of spontaneously labeled vesicles distinguished between the axon, the dendrite and the soma (for soma vs. dendrite two-sample Kolmogorov-Smirnov test: *p*<0.001; for soma vs. axon two-sample Kolmogorov-Smirnov test: *p*<0.001; for axon vs. dendrite two-sample Kolmogorov-Smirnov test: *p*=*ns*). **E** Fractions of the reserve and recycling pool normalized on the total pool. Boutons were separated into small, medium and large synapses according to their total pool size (Kruskal-Wallis test: *χ*211, 1510=20.62, *p*<0.05).


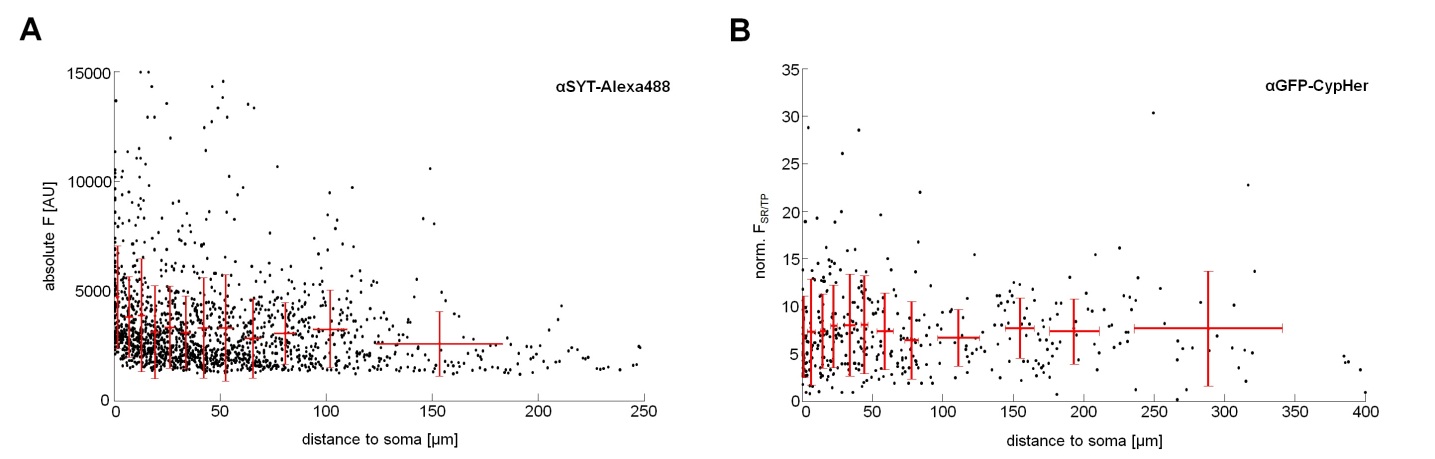


Fig.S5: Relationship between the distance from the soma and spontaneous release. **A** Correlation between the number of spontaneously fused vesicles labeled with αSYT-Alexa488 and the distance to the soma of the respective synapse (Spearman`s rank correlation coefficient *ρ*=-0.80; *p*=0.002). **B** Correlation of the SR to TP ratio and the distance to the soma (Spearman`s rank correlation: *p*=*ns*). Spontaneous released vesicles were detected using αGFP-CypHer5E^TM^ antibody in spH transfected neurons. TP were determined by NH_4_^+^ application. For group wise comparison, synapses were grouped in twelve equally sized bins and averaged. Error bars indicate STD.
